
JOURNAL INFORMATION
==============================
NLM Title Abbreviation: Atlanta Med Surg J
NLM Journal ID: 0001010

Atlanta Medical and Surgical Journal

Publisher: Biomedical Journal Digitization Project

ARTICLE INFORMATION
==============================
PMCID: PMC8823840
PMID: 35823958
Article version: 1
Subjects: Editorial and Miscellaneous

Pruritus Pudendi Successfully Treated by Sulphite of Soda

Frizell Samuel B. M. D. Grangerbury, Ohio

Print publication date: 1867 Feb
Volume: 7B
Volume Series: New Ser
Issue: 12
First page: 576
Last page: 576
License: This work is free of known copyright restrictions. For more information, please see PMC Back Issue Digitization.
License URL: https://creativecommons.org/publicdomain/mark/1.0/

==============================

*Pruritus Pudendi successfully treated by Sulphite of Soda.* By Samuel B. Frizell, M. D., of Grangerbury, Ohio.

In September, 1866, I was consulted by a lady suffering from pruritis pudendi following menstruation—accompanied with great irritation and much pain.

Having read of the influence of sulphite of soda on sycosis menti, the idea suggested itself to me of trying the same in this case. I accordingly prescribed for her the following local application: Sodæ sulphis ʒj, aquæ ʒiij, glycerinæ ℥j, M., which was to be used quite often. In three days no trace of the disease was apparent.—*American Journal of Med. Science.*

---

### MASSEY & HERTY,

Whitehall Street,

ATLANTA, GEORGIA,

WHOLESALE AND RETAIL DEALERS IN

**Pure Drugs, Chemicals, Patent Medicines,**

Perfumeries, Toilet Articles, Stationery, Cigars

**Wines, Liquors, Dye-Stuffs, Paints,**

OILS, BRUSHES, GLASS, PUTTY, &C.

Coal Oil and Lamps, Machine and Tanners' Oil always on hand.

☞ *Will duplicate all Orders made South.*
